# Autonomy-Supportive Teaching in Medicine: From Motivational Theory to Educational Practice

**DOI:** 10.15694/mep.2021.000117.1

**Published:** 2021-05-10

**Authors:** Adam Neufeld

**Affiliations:** 1 University of Calgary

**Keywords:** autonomy-supportive teaching, controlling teaching, motivation, basic psychological needs, medical education

## Abstract

This article was migrated. The article was marked as recommended.

**Introduction:** Promoting intrinsic motivation in medical learners is key for maximizing their engagement, performance, and well-being. Accordingly, methods of teaching and giving feedback focused on enhancing intrinsic motivation (i.e. autonomy-supportive approaches) are becoming increasingly recognized in medical education. However, these methods seem to be limited by a lack of understanding around what autonomy-support means and how to put it into practice.

**Approach:** Grounded in Self-Determination Theory (SDT), this article defines autonomy-supportive versus controlling teaching, provides a rationale for its importance in medical education, addresses common misconceptions and potential challenges surrounding autonomy-supportive teaching in medicine, and outlines specific examples of ways to engage in it, in both clinical and non-clinical settings.

**Conclusion:** Overall, this paper highlights how simple, flexible, and beneficial it can be to utilize an autonomy-supportive style in one’s day-to-day interactions with medical learners, to stimulate their intrinsic motivation.These strategies can be applied in practically any setting and at all levels in medical education, through regular practice and reflection.

## Introduction

Promoting medical learners’ intrinsic motivation is key for maximizing their engagement, performance, and well-being. In view of this, methods of teaching and giving feedback focused on enhancing intrinsic motivation (i.e. autonomy-supportive approaches) are beginning to gain some traction in medical education. However, practical guidelines on autonomy-supportive teaching strategies remain relatively scarce, and there is often confusion about what autonomy-support truly entails ( Ten Cate, Kusurkar and Williams, 2011; Baldwin *et al.,* 2012; Kusurkar and Croiset, 2015). This can hinder medical educators’ ability to be autonomy-supportive, as well as perpetuate teaching strategies that inadvertently undermine learners’ intrinsic motivation and well-being. This gap highlights the need for more clarity around the concept of autonomy-supportive teaching in medical education-where challenging environments and teacher interactions play an integral role in learners’ development and ability to flourish.

The aims of the present paper are to fill this gap by illustrating how simple, flexible, and effective it is to adopt an autonomy-supportive style in one’s medical practice and day-to-day interactions with medical learners. To that end, the current article: 1) covers a brief overview of Self-Determination Theory (SDT), 2) defines and discusses autonomy-supportive versus controllingteaching, 3) provides a rationale for why these concepts are highly relevant to both medical learners and teachers, with references to the extant medical education literature within SDT, and 4) outlines specific examples of strategies that support versus thwart medical learners’ intrinsic motivation and well-being. Note that, for the purpose of this paper, medical “educator” or “instructor” refers to those in positions of authority and trust (e.g. medical teachers, preceptors, residents, and attending physicians), and medical “learner” refers to students and residents.

## Objective 1: Provide a Brief Overview of Self-Determination Theory (SDT)

SDT represents a broad framework for the study of human motivation, development, and well-being ( Ryan and Deci, 2000, 2017). It is an organismic theory which posits that human beings are active creatures with evolved tendencies towards exploration and learning, taking on challenges, cultivating social relationships, and integrating new experiences into a coherent sense of self. For these inherent drives to persist and function optimally, SDT states that people universally require ongoing support for three basic psychological needs-autonomy, competence, and relatedness ( Ryan and Deci, 2000, 2017). Autonomy is the need for agency and to feel that one’s choices are volitional (i.e. that one’s behaviours and activities are congruent with one’s integrated sense of self) versus feeling pressured or controlled. Competence is the need to feel capable and effective, which is fuelled by overcoming challenges and mastering skills. Relatedness is the need to feel a sense of connectedness and belonging with others, which is deeply satisfied when one feels understood, valued, and respected.

As SDT concerns itself with the social conditions that promote versus forestall people’s motivation (which is thought to reflect varying levels of need fulfilment), it recognizes that people are moved to behave for a variety of different reasons. As such, SDT focuses less on the strength and uniquely more on the type and quality of motivation. More specifically, SDT emphasizes that motivation exists along a relative continuum of autonomy-ranging from amotivation (which requires no internalization and is therefore non-autonomous), to various types of extrinsic motivation (which differ in their degree of internalization and correlate with more or less autonomous behaviour regulation), to intrinsic motivation (which is considered most autonomous, as it arises internally). Accordingly, it is SDT’s view that, while both extrinsic and intrinsic types of motivation can be powerful drivers of behaviour, intrinsic motivation is more self-determined and thus more conducive to optimal functioning, vitality (feeling alive and having energy available to the self) and psychological well-being ( Nix *et al.,* 1999).

**Figure 1.**
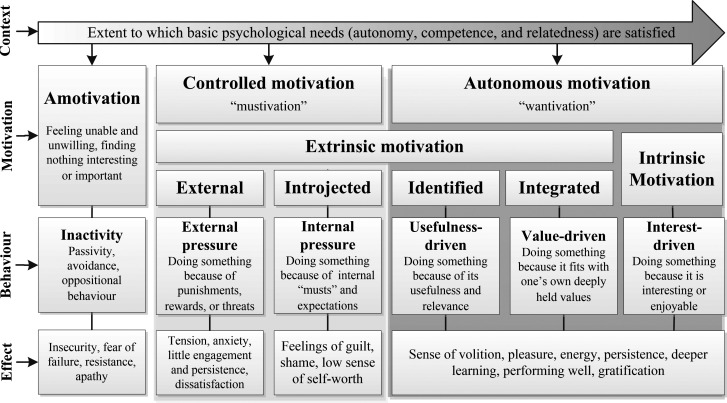
SDT’s motivation continuum

Essentially, SDT views affordances and barriers to psychological need fulfilment as fundamental for self-actualization and well-being, in that greater need satisfaction facilitates more self-determined or autonomous motivation and need frustration leads to more non-self-determined or controlled motivation ( Ryan and Deci, 2017). In support of this, many studies show that environments that serve these basic needs tend to promote greater behavioural persistence and healthier psychological outcomes (e.g. resilience, revitalization, and flourishing) compared to environments that hinder them, which tend to provoke ill-being (e.g. stress, maladjustment, and psychopathology) (e.g. Bartholomew *et al.,* 2011; Vansteenkiste and Ryan, 2013). While numerous other need-related mini-theories exist within SDT (which are beyond the scope of the present paper), to help illustrate the theory as a whole, I present an adapted model of SDT’s motivation continuum (see Figure 1, Source: Visser, 2017). It uniquely captures all of SDT’s elements in one picture-the level of need fulfilment and the corresponding types of motivation (i.e. degree of internalization), reasons for behaviour, and psychological consequences.

(adapted with permission from Visser, C. F., based on work by Deci and Ryan (2000) and others in the field of SDT).

The dynamics of basic psychological need fulfilment and their effects on motivation have been investigated in many different domains (e.g. in organizations, classrooms, healthcare, sports, families, and cultures). In an educational context, research shows that intrinsically motivated learners tend to be more engaged and experience deeper learning, stronger academic performance, and greater feelings of purpose and psychological well-being, compared to extrinsically driven learners ( Ryan and Stiller, 1991; Boggiano *et al.,* 1993; Black and Deci, 2000; Vansteenkiste *et al.,* 2009). Research in the health professions and in medical education is complimentary ( Kusurkar *et al.,* 2013; Orsini, Binnie and Tricio, 2018; Neufeld and Malin, 2020b). Nevertheless, SDT research within medical education is still considered relatively scarce, and while various autonomy-supportive practices (e.g. Kusurkar *et al.,* 2011), teaching strategies (e.g. Neufeld and Malin, 2020a), and misconceptions surrounding autonomy-support (e.g. Kusurkar and Croiset, 2015; Neufeld and Malin, 2020a) have been discussed in the extant literature, these articles are not comprehensive and have several noteworthy limitations.

Kusurkar *et al.* ( 2011, 2015) focused primarily on autonomy-supportive classroom strategies and structural considerations in medical education (e.g. types of teaching settings, vertically integrated curricula, the concept of entrustable professional activities, and various elective opportunities for students), that are insightful and practically useful for medical educators. However, these articles did not delve into clinical environments, which are equally important in a learner’s medical education. Moreover, they did not cover specific controlling actions, which many medical educators may wish to better understand and avoid. These are important considerations, given clinical settings (which are often quite different and less predictable environments than classrooms) may not always be as malleable as classroom teachers, in supporting medical learners’ basic needs. In view of this, the present paper focuses evenly on autonomy-supportive and controlling teacher actions and how they might apply, both within the classroom and in clinical milieus-where many physician educators spend a great deal of their time, teaching.

## Objective 2: Define autonomy-supportive and controlling teaching

### Autonomy-supportive teaching styles

Essentially, autonomy-supportive teaching means working from learners’ perspectives to promote their engagement, sense of personal responsibility, and feelings of competence. In other words-using a humanistic approach to pedagogy that helps medical learners feel capable and involved in the educational partnership ( Williams and Deci, 1998). In a medical education context, the behaviours most central to autonomy-supportive teaching concern the provision of choices (e.g. about how to behave in one’s role), sharing useful information that is necessary to make wise decisions (e.g. in clinical scenarios or with career-related planning), giving a meaningful rationale for suggested actions (i.e. to help medical learners identify the value and facilitate self-endorsement), acknowledging feelings about behavioural options, both positive and negative (i.e. to help learners feel like stakeholders), and encouraging learners to self-initiate and persist in their efforts (i.e. to find their own solutions to various challenges, rather than instructors insert themselves too much or give learners the answers) ( Williams and Deci, 1998).

An important yet often overlooked aspect of autonomy-supportive teaching is supporting learners’ psychological needs for competence and relatedness. There is a reason why good teachers will often kickstart their lessons with light-hearted introductions or personal anecdotes, and why easier cases or problem-solving challenges invariably precede tougher ones. Whether deliberately or not, they do this because it tends to promote more active audience participation (in other words-more autonomous engagement). According to SDT, this occurs because it appeals to learners’ feelings of relatedness and competence, which in turn, supports their autonomy. In other words, people prefer to engage in activities they can understand and master, and are more likely to listen and adopt information from others they identify with ( Niemiec and Ryan, 2009). Hence, while SDT views autonomy as the anchor (in that environmental supports for autonomy tend to facilitate people’s ability to fulfil their other basic psychological needs for competence and relatedness), all three needs are synergistic, and supporting each is essential to being autonomy-supportive and stimulating intrinsic motivation.

### Common myths about autonomy-supportive teaching

Given this definition of autonomy-support, it is important to dispel a few common misconceptions about what it means to engage in autonomy-supportive behaviours. The first is that autonomy-supportiveness is more of a trait, rather than a skill. As Kusurkar and Croiset (2015) highlight, however, being autonomy-supportive means much more than that-it represents a fundamental change in the way of thinking and working in medical schools (i.e. to foster more autonomy among all those involved in education). Practically speaking, this translates into recurrent questioning of one’s methods and adapting of one’s techniques, in order to best suit the persons and situations involved. As such, being autonomy-supportive is considered a skillful task that can most certainly be learned and developed ( Reeve, 2002)-the first step being that educators ‘unlearn’ various controlling behaviours ( Kusurkar and Croiset, 2015).

Another common misconceptions is that supporting medical learners’ autonomy equates to giving them independence. While providing independence (i.e. freedom from outside support) may promote autonomy (i.e. feelings of volition in one’s own behaviours and goals) in some situations, autonomy and independence do not represent the same thing ( Ten Cate *et* *al.,* 2011). A clear example of this is the “see one, do one, teach one” analogy-a traditional approach to clinical teaching in Medicine which may be misconstrued as autonomy-supportive, when in fact it is more likely to be controlling (given its prescriptive nature). Although this method of teaching may support greater independence, a key point to recognize is that medical learners can be autonomous in their choice to be relatively dependent in certain situations (e.g. in learning a new skill or procedure). This makes adhering to a strict “ *see one, do one, teach one*” approach potentially inappropriate in various situations ( Neufeld and Malin, 2020b). The takeaway is that each medical learner and their learning needs are uniquely different, and while some may perceive independence as desirable, others may perceive it as pressure (e.g. to perform a task they do not feel intrinsically ready for).

Finally, it is a commonly held belief that, for a medical educator to be autonomy-supportive, it is a prerequisite that they solicit and/or cater to every individual learner’s perspectives or input, which by default would make it impractical in many settings (e.g. in large groups, or when teaching topics that involve less learner participation, such as gross anatomy) and force instructors to modify curriculum or sacrifice the quality of their teaching. While these misinterpretations are understandable, they could not be further from the truth. In fact, Kusurkar *et al.* (2011) provide a dozen ways that medical educators can be autonomy-supportive in the classroom (e.g. by providing emotional support and structured guidance, setting optimal levels of challenges for learners, and explaining the rationale for learning materials), which they further suggest may also apply in large group teaching settings, clinical encounters, and problem-based learning scenarios. Other studies are complimentary, highlighting that teachers tend to become more autonomy-supportive once they realize how simple it actually is ( Reeve and Cheon, 2016), and that being autonomy-supportive mutually benefits the teacher-i.e. in promoting greater feelings of self-efficacy, job satisfaction, well-being, and harmonious passion for teaching ( Cheon, Reeve and Vansteenkiste, 2020). The one caveat I might add is simply that, in certain situations, it is possible that some strategies to support learners’ autonomy may be more or less effective and thereby require a bit of mindfulness and flexibility, on the part of the medical educator (see Objective 4 below, for tips and examples).

### Controlling teaching styles

To fully capture what autonomy-supportive teaching entails, it is critical to understand what the opposite of an autonomy-supportive style is-that is, a controlling one, in which individuals in positions of authority (e.g. instructors, senior residents, practicing physicians) introduce, into learning environments, the use of external controls, overly close monitoring or supervision of learners, and/or evaluations and feedback that center around rewards and punishments. One example of this would be insisting that learners must study certain concepts because, “They will be tested on your module exam, which requires passing, in order to satisfy the program requirements.” Research shows these practices tend to reflect external pressures on teachers ( Ryan and Brown, 2005), as well as educator beliefs that motivation is better shaped through external contingencies of reinforcement, rather than by facilitating learners’ genuine interests (i.e. ‘wantivation’) in their learning. As Niemiec and Ryan (2009) point out, this creates the unfortunate and self-fulfilling prophecy that plagues many teaching environments, in which controlling conditions replace learners’ feelings of joy, enthusiasm, and interest in their learning, with experiences of anxiety, alienation, and boredom. As a result, learners become disinterested and teachers resort to externally regulating students’ behaviour in order to force learning to happen (i.e. through ‘mustivation’).

Of course, while some medical educators may find themselves operating on either end of the controlling versus autonomy-supportive spectrum, many will doubtless fall somewhere in between-that is, whom are neither controlling, nor particularly attuned to learners’ psychological needs for self-determination. For instance, many physicians may welcome new learners yet provide them with little structured guidance or feedback-to help them feel capable of success. Moreover, medical educators may be polite and professional but show little empathy or interest in their learners (i.e. in their background, level of knowledge, goals, and challenges), which can lead them to refrain from giving learners responsibility or freedoms (e.g. to see patients alone)-out of a lack of awareness and trust. This may be expressly true, if the physician perceives their own sense of control and schedule might be threatened (i.e. due to a learner potentially slowing them down). While generally well-intentioned and not overtly controlling, these passive or constraining approaches to supervision can send the message that learning is not a high priority. As a result, medical learners-whose basic needs are neither supported nor necessarily frustrated-may feel somewhat resigned and demotivated about their learning.

In medical education, there is still a surprising reliance on ‘carrots and sticks’ approaches to pedagogy. Take “pimping” (otherwise known as “hazing”) for example-slang terms for an aggressive style of questioning physicians commonly use, in order to push medical learners and test their clinical knowledge, when on the hospital wards or in clinic-most often in front of patients and other learners. Indeed, this can be a powerful motivator for medical learners, given the initial response it tends to provoke-e.g. anxiety, humiliation, and/or guilt, followed by a strong urge to study harder (to avoid similar feelings in the future). However, because this method motivates learners to study out of ego-defense and feelings of obligation or fear (i.e. through introjected motivation) versus by recognizing the importance and having a genuine interest in learning the content (i.e. via identifiedand integratedtypes ofmotivation), SDT would view this method as controlling ( Palmer and Malin, 2019). As such, it is considered less conducive for deep learning, persistent efforts, and not to mention-well-being. This example highlights precisely why SDT concerns itself less with the quantity of one’s motivation (which can be high) and focuses more on the quality of one’s motivation (which can simultaneously be low).

Another controlling aspect of a modern-day learners’ medical education is mandatory wellness curriculums-formalized venues that are routinely being adopted in medical schools, which focus on fostering medical learners’ resilience and mindfulness, to help them cope more effectively with stressors in the learning environment ( Wasson *et al.,* 2016; Velez *et al.,* 2019). While prevalent among medical institutions, these policies tend to force (rather than invite) learners to participate, which studies show can hinder satisfaction ( Aherne *et al.,* 2016) and actually add to feelings of distress and burnout for medical learners ( Ishak *et al.,* 2013; Squiers *et al.,* 2017). Medical student, Senger (2019), captures this paradox in his CanadiEM commentary, saying, “...it is ironic that a profession that emphasizes patient autonomy, so strongly, refrains from giving students the autonomy to determine which wellness activities are most congruent with their interests and goals.” Through an SDT lens, mandatory wellness training is more likely to create apathy and hinder engagement because it thwarts autonomy, by externally managing something medical learners are already intrinsically motivated to do ( Williams and Deci, 1998)-that is, to maintain their own health and well-being, in what ways and with what free time their studies permit them.

## Objective 3: Exemplify the value of supporting medical learner autonomy

For medical learners, transitioning from the classroom into clinical training can be quite a stressful experience ( Hojat *et al.,* 2009). At this stage, medical learners are thrusted into unchartered territory-where adjusting to new clinical environments can threaten their autonomy and other basic psychological needs ( Neufeld and Malin, 2020b). An important consideration in this matter is the dynamic interplay between learner and environment and how the learning climate facilitates or disrupts integration processes that underlie need fulfilment and well-being. For example, many learners may feel that their new schedules leave them no time for themselves, or that their medical training is a “grind” and not enjoyable anymore, which can erode empathy towards patients ( Hojat *et al.,* 2009). Medical learners may also compromise their own learning needs (e.g. for teaching, feedback, and support) if they fear it might interfere with patient care, if they sense the environment to be judgmental, or if they perceive their supervisors are stressed or not approachable. These feelings (which relate to perceptions of autonomy, competence, and relatedness) are critical from an organizational psychology standpoint (i.e. with work satisfaction), and in optimizing patient outcomes, with respect to the quality of care that medical learners provide ( Williams and Deci, 1998; Black and Deci, 2000).

In their longitudinal study on patient-interviewing, Williams and Deci (1996) found that medical learners who perceived their instructors were more autonomy-supportive reported higher levels of autonomous self-regulation, increased perceived competence, and demonstrated greater internalization of the biopsychosocial values they were taught, surrounding patient interviewing ( Williams and Deci, 1996). Notably, when a follow-up study was conducted two years later, students who self-regulated more autonomously toward patient interviewing were, in turn, rated as more autonomy-supportive by their standardized patients, implying their adoption of the values they learned remained intact. Relatedly, other studies showed that, when medical educators in internal medicine and surgery were more supportive of medical clerkship students’ autonomy, it engendered greater feelings of competence and intrinsic motivation to pursue those careers, compared to instructors in those specialties, that were more controlling ( Williams *et al.,* 1994, 1997). Put together, these studies demonstrate that autonomy-supportive instructors and learning environments promote core values that enhance learner interest, autonomous self-regulation, and competence fulfilment-qualities that predict higher well-being and lower burnout among physicians ( Shanafelt, Sloan and Habermann, 2003).

Fast forward several decades and new and exciting SDT research continues to emerge in the health professions and medical education fields. For instance, research has demonstrated that, among students, more autonomous motivation and feelings of need fulfilment were associated with greater meaning and satisfaction in life, better study effort and academic performance, and less work-related exhaustion ( Kusurkar *et al.,* 2013; Bailey and Phillips, 2016; Orsini, Binnie and Wilson, 2016). Similarly, other studies conducted among Canadian medical students found that, when medical learners perceived their workplace and instructors were more autonomy-supportive, it related to higher resilience and greater psychological well-being ( Neufeld and Malin, 2019, 2020b). Additionally, psychological need fulfilment, more than mindfulness and resilience, was found to relate to medical students’ perceived stress in medical school ( Neufeld and Malin, 2020c) **,** suggesting that their stress levels may reflect or vary as a function of their motivational needs. These findings align with research within SDT ( Brown and Ryan, 2003; Niemiec, Ryan and Brown, 2009; Weinstein, Brown and Ryan, 2009; Weinstein and Ryan, 2011) and have potentially significant implications in medical education, particularly given the high level of physician stress and burnout in the profession.

In sum, there is a growing body of empirical evidence supporting that basic psychological need fulfilment is highly important to the quality of motivation and mental health of medical learners, at both undergraduate and postgraduate levels. Despite this, however, many gaps continue to exist between research and policy in medical education-i.e. between what studies tell us about intrinsic motivation and what regulations are instituted and upheld in medical education (e.g. in curricular design, delivery, and assessment, in the development and implementation of wellness interventions, in the way adult learners are taught and supervised during their medical training, and how their progress is monitored and evaluated by medical educators and programs). As Niemiec and Ryan (2009) aptly put it (p. 140), drawing from work by Deci and Ryan (2002), “To the extent that administrators and policy makers fail to consider the motivation of both teachers and learners alike, and instead rely on controlling contingencies to produce ‘accountability’, the more all those involved in the process will suffer decrements in motivation and learning outcomes.”

Ultimately-no matter what the level or structure of any medical program-by being more aware of these basic psychological needs and how to support and not thwart them (particularly for medical learners, but also amongst everyone else involved in the educational process), programs and educators may be better positioned to help close these gaps and promote excellence and humanism in medical education-i.e. in ways that enhance the development and achievement of medical learners, without compromising their self-determination and psychological well-being ( Williams and Deci, 1998; Patrick and Williams, 2009; Kusurkar, Croiset and Ten Cate, 2011; Baldwin *et al.,* 2012; Neufeld and Malin, 2020b).

## Objective 4: Outline specific autonomy-supportive and controlling teaching strategies

While medical educators have an undeniable role in shaping learners’ medical education and psychological need fulfilment, how best to support and teach medical learners (without unknowingly causing psychological harm) may not always be apparent or straightforward. This may be especially difficult, when trying to balance various other competing responsibilities, such as clinical care management (i.e. triaging patients, consulting with other staff members, and maintaining safe and high quality patient care) and administrative tasks (e.g. billing, charting, and ensuring orders, admissions, and discharges are dealt with, appropriately). Hence, the clinical environment could be considered less stable and predictable than the classroom and therefore conflict with or distract from teaching and involving medical learners, in ways that support their basic needs for self-determination.

Medical educators may also find it challenging to address need-thwarting aspects of clinical medicine, which may be felt to have systemic origins that are less remediable (e.g. daunting schedules and traditionally-held workload expectations). Moreover, supporting medical learners’ basic psychological needs may be perceived as arduous-for example, on fast-paced medical services (e.g. acute care surgery) that may offer little time for individual supervision or personalized feedback, when there are many medical learners to supervise (e.g. on a busy internal medicine teaching unit), or if there are learners at multiple different stages of training (e.g. pre-clinical medical students, new versus experienced clerkship students, and junior and senior residents). By extension, medical educators may mistake learners’ psychological needs for luxury ingredients (i.e. for when times are good), when in reality they are even more critical to pay attention to, during times of stress ( Rigby, 2020; Neufeld and Malin, 2020d), such as in educational transitions and adjusting to bustling clinical environments ( White, 2007; Dunham *et al.*, 2017).

These are all legitimate challenges that many medical educators may face, and merely being more interpersonal and autonomy-supportive does not necessarily solve all of them. That said, however, Neufeld and Malin (2020b) propose a number of relatively simple actions that medical teachers can take, which can make a large difference in shaping the experiences of medical learners-that is, in promoting or undermining their perceptions of autonomy, competence, and relatedness, in medical school. These autonomy-supportive versus controlling teacher actions and communicative techniques, which are summarized and elaborated on below, are well-supported by SDT research within the education ( Reeve and Jang, 2006; Reeve *et al.,* 2014) and healthcare fields ( Teixeira *et al.,* 2020).

### Autonomy

According to Neufeld and Malin (2020b), medical educators can support medical learner autonomy-regardless of their level of medical training or discipline-by providing them with choices and options (within the rules or confines of the situation), by trying to acknowledge and understand their viewpoints (including those of negative affect, which can help identify potential externalized or partially internalized pressures or expectations), by providing structure and guidance with clear objectives, offering meaningful rationales for behaviours (to highlight and reinforce motives that could form the basis for autonomous motivation), and by challenging learners to self-initiate, experiment, and discover their own solutions to various questions or problems (e.g. asking medical learners what their impressions and theoretical plans might be, for managing various clinical conditions, rather than simply taking the lead and deciding). Many of these are feasible on short time and with teams of medical learners at various levels of experience.

Conversely, Neufeld and Malin (2020b) note that medical educators will tend to undermine medical learner autonomy by micromanaging, overpraising and spoon-feeding (which may be interpreted as patronizing or belittling), by being dismissive or defensive (e.g. in providing and receiving feedback), and by using controlling (“you must,” “you need to,” and “you should”) versus autonomy-supportive (“can you,” “you might,” and “could you”) language with learners. These actions (which are thought to block organismic growth, prevent self-actualization, and constrain authentic behaviour) may apply across a range of different situations in medicine-e.g. in the classroom, during small and large-group teaching, in team-based patient encounters (e.g. rounding), when giving learners feedback and evaluations, and in corresponding with medical learners (e.g. when reviewing cases over the phone, when sending emails, and in developing or reforming course or clinical rotation syllabi).

### Competence

Competence, which is pivotal for autonomy and resilience, might be the most threatened psychological need during a learners’ medical education-especially in clinical settings, where demands can be high and environments less convivial ( Neufeld and Malin, 2019). From undergoing constant evaluations and high stakes tests (e.g. national board medical exams), to living up to perceived expectations and regularly having to demonstrate skills in front of others (staff, peers, and patients), medical learners can experience significant issues of self-confidence and distress during their medical education ( Blanch *et al.,* 2008; Villwock *et al.,* 2016). Nonetheless, while some of these aspects may be engrained in a learner’s medical curriculum, Neufeld and Malin (2020b) highlight that medical educators can mitigate some of these pressures, by using various competence-supportive approaches.

To support medical learners’ feelings of competence, they recommend providing-as much as possible-structured learning environments with clear expectations and guidance (in both experiential or process-based realms and with respect to desired outcomes), offering constructive, relevant, and informational (i.e. non-judgmental) feedback to learners, and perhaps most importantly, setting optimal (developmentally appropriate) levels of challenge for learners-i.e. not putting them into (or withholding them from) situations, or teaching them, at levels too far beyond or below their capabilities. An important part of successfully navigating these approaches-particularly if a learner may be struggling with certain concepts or skills-is promptly identifying likely barriers to facilitating that individual’s success or behavioural change (i.e. based on their previous attempts), explaining how to overcome them (which can relieve stress, promote confidence, and reinforce existing skills), and promoting self-monitoring (which provides structuring information and fosters self-awareness). Finally, medical educators may help support learners’ perceived competence by exploring ways of dealing with stress or failure, in performing their jobs effectively (i.e. sharing information on ways to be resilient and manage pressure from others or themselves).

In contrast, medical educators may undermine learners’ ability to fulfil their need for competence by providing minimal structured guidance, or feedback that is irrelevant (not goal-oriented), unclear (unspecific), or personally evaluative (versus process-driven). Part of this entails that medical educators pay attention and check in regularly with their medical learners, so they can adjust things (e.g. the level of challenge, supervision, guidance, and feedback) when they notice their learner(s) are over- or underwhelmed with the task(s) at hand. Hence, while not taking stock of medical learners’ perspectives, performance, and well-being may not be controlling (in that it does not assert power or introduce contingencies), because it prevents identification of goals that are realistic, meaningfully challenging, and achievable, it can forestall competence fulfilment (and thus, autonomous motivation). Again, the use of intimidating tactics such as “pimping” (belittling and questioning learners beyond their level of knowledge) or cookie cutter (e.g. “see one, do one, teach one”) methods of teaching, which tend to be more controlling and outcome- versus person-focused, also fit into this category and thus are generally considered unsupportive of medical learners’ need for competence and intrinsic motivation.

### Relatedness

Finally, relatedness is a key need for learners-especially when it comes to identifying with teachers and integrating new knowledge. According to SDT, people internalize and integrate values more, when they come from individuals or groups (e.g. parents, teachers, mentors) they wish to assimilate with or emulate ( Ryan and Deci, 2017)-in this case, with medical learners, from their physician role models. In support of this, Neufeld and Malin (2020b) found that feelings of relatedness, over autonomy and competence, emerged as the strongest mediator of the relationship between medical learners’ perceptions of instructor autonomy-support and their psychological well-being. These findings suggest that medical educators may support learner autonomy best, by prioritizing their feelings of relatedness. While the specific reasons for this finding were not assessed in the aforementioned study, making the connection seems intuitively simple-there is psychological safety in relatedness and being in a medical learners’ shoes can often feel like walking on eggshells ( Gold, Rosenthal and Wainberg, 2019).

As the authors of the latter study point out, fostering psychological safety does not imply that medical instructors should duck difficult conversations with medical learners or avoid exposing them to challenging or potentially anxiety-provoking learning environments, as these can be critical for growth. From an SDT perspective, it simply means considering medical learners’ human psychological needs, so they can navigate such experiences more authentically and adaptively, in a way that supports their autonomy. In effect, doing this may allow medical learners to experience not only more relatedness (i.e. with their colleagues and supervisors), but also an enhanced sense of freedom to be their true selves and show more vulnerability (e.g. to admit perceived weaknesses)-something that is considered a fundamental process for genuine learning and personal development to occur ( Ryan and Deci, 2017).

To support medical learners’ perceptions of relatedness, Neufeld and Malin (2020b) suggest showing interest in learners, encouraging them to ask questions, and using empathic listening (which displays respect and promotes trust and collaboration). Additionally, they recommend investing time in getting to know medical learners (i.e. their goals, challenges, and interests), trying to have a warm and approachable demeanor of unconditional regard (expressing positive support regardless of success or failure), using shared decision-making with learners (e.g. in clinical situations and in feedback and evaluations), making learners feel valued (e.g. welcoming them, getting to know their names, delegating important tasks to them, involving them in team-related healthcare decisions and planning), and demonstrating empathy and humility (e.g. acknowledging limitations and/or mistakes). In contrast, medical educators can undermine their medical learners’ sense of relatedness by being inaccessible or standoffish, showing disinterest in learners, dismissing their perspectives and contributions, and/or by giving harsh, judgemental, or impersonal feedback. Relatedly, it is also important to note that, when communicating with learners, to promote more intrinsic motivation, the tone or prosody that educators use may be just as important (if not more so) than the content of the feedback itself ( Weinstein, Zougkou and Paulmann, 2018).

## Tying it all together

As this paper highlights, each of the three basic needs are tightly intertwined and integral to psychological health, development, and well-being. Hence, contexts that support versus thwart these needs-especially simultaneously-may be particularly salient for a learner, in their self-regulation, performance, and attitudes towards their learning. One such area, where supporting learners’ autonomy, competence, and relatedness may be especially important, is in the way instructors provide feedback ( Mouratidis, Lens and Vansteenkiste, 2010). This can be a real source of stress for medical learners, given the high performance expectations and pressures they will often face in medical school (both real and imagined) and the between-instructor variability that tends to occur, in this area ( Bowen, Marshall and Murdoch-Eaton, 2017).

Typically, medical educators will complete evaluations toward the end of clinical sessions, shifts, or rotations, according to both intuitive (i.e. gestalt) and prescribed performance standards (e.g. on an expectations-based scale, ranging from “below,” to “beginning to meet,” to “meets,” to “exceeds”). Unfortunately, it is my observation that many physician instructors will generically default to “meets expectations” across the board, will not necessarily seek to know or actively involve their learners in their own evaluations, and for actionable suggestions, write something along the lines of, “Continue reading and asking questions” or simply, “No concerns.” Quite often, they may also focus strictly on intellectual versus affective aspects (i.e. medical knowledge) without acknowledging the many nuances and humanistic qualities or skills that learners may possess and demonstrate (e.g. empathy and awareness, a positive attitude and demeanor, strong communication and collaboration, professionalism and leadership, and maturity and reliability). Thus, while a simple “meets expectations” evaluation may temporarily relieve a medical learner of their performance apprehensions, and is not necessarily controlling per se, it also does little or nothing to stimulate intrinsic motivation or promote growth and development.

In contrast, an autonomy-supportive preceptor might take some time to sit down with their medical learner, discuss any cases the learner may have found particularly interesting or challenging, and inquire about their learners’ thoughts and feelings surrounding their own experiences and performance. Following this, the preceptor might propose strengths and potential areas for improvement, explain the rationale for their suggestions and feedback, and encourage learners to continue in their efforts to become more proficient, while linking the value of the clinical learning and specified feedback to the learners’ individual situation (e.g. perceived level of competence, set goals, and/or career-related interests). To take it one step further, the instructor may ask the medical learner for feedback, in return-i.e. on how they can improve as a medical teacher. Beyond exemplifying a growth mindset and commitment to lifelong learning, this also shows humility and promotes positive feelings of collaboration.

Between the two scenarios, the latter approach would facilitate stronger, lasting motivation to persist and self-evolve (even if the learner gets a few “below” or “beginning to meet” evaluations), because it promotes autonomy, competence, and relatedness. In turn, it opens the door for less defensive coping (i.e. in response to constructive feedback), more autonomous self-regulation and feelings of competence, and greater overall well-being ( Ryan and Deci, 2017). Rather than by being controlling, the instructor impressed, upon their medical learner(s), the value of certain knowledge and attitudes, by affirming what they did well, using a tone of understanding, and empowering them-that is, by encouraging and challenging learners in ways that are congruent with their developmental level and intrinsic goals, values, and/or career interests, which supports their basic psychological needs ( Deci and Ryan, 2000; Vansteenkiste, Lens and Deci, 2006; Baldwin *et al.,* 2012).

## Conclusion

Guided by SDT, this article highlights some of the benefits, misconceptions, and potential challenges of adopting an autonomy-supportive teaching style in medical education. As much as possible, this involves creating a positive, structured, and non-controlling learning environment that supports medical learners’ basic psychological needs for autonomy, competence, and relatedness. By being autonomy-supportive (versus controlling), which promotes learners’ ability to internalize and integrate the values associated with their learning, it helps to stimulate a sense of meaningfulness and intrinsic motivation. As a result, medical learners become more active agents in the learning process and can respond more adaptively to their surroundings. In the medical profession-where processing emotionally charged events is critical, rates of distress and burnout are high, and psychological qualities like mindfulness and resilience are considered especially valuable-finding ways to support medical learners’ basic psychological needs is a noble and time worthy pursuit.

My hope in extending prior work in this area was threefold. Firstly, by focusing not just on autonomy-supportive actions but also controlling methods of teaching that undermine intrinsic motivation, it may help medical educators to better recognize and avoid methods of pedagogy that can cause psychological harm. Secondly, by discussing SDT’s utility beyond the classroom and in various clinical contexts, it may translate into richer, more rewarding teacher-learner experiences, and consequently, higher quality patient care. Finally, by addressing several common misconceptions about autonomy-supportive teaching and acknowledging potential limitations of its use (and not just its benefits) in medical education, it can help medical educators, from a broad range of backgrounds, to more easily translate principles of SDT into their leadership and teaching practices. It is in these settings that applications may be highly consequential to medical learners’ motivation, development, and psychological well-being, in their quest to become physicians.

In closing, Nobel prize winning physicist and professor, Albert Einstein, once said, “It’s the supreme art of the teacher to awaken joy in creative expression and knowledge.” Patrick & Williams (2009) echo this sentiment in their petition of encouragement to medical educators: to consider not just what but how they teach, and to model their styles after those of blues artists and poets, who frequently repeat their lyrics to enhance their listeners’ experience and integration of the intellectual *and*emotional messages(p.184). In essence, what both are implying is that, while control may lead to compliance, a learning climate that supports autonomy is what leads to authentic engagement, happiness, growth, and well-being. Medical educators who abide by this principle-who weave more humanism into the fabric of their practices-will not only bolster their learners’ need fulfilment and joie de vivre, but also that of their learners’ future patients and learners too.

## Take Home Messages

• In medical education, autonomy-supportive teaching relates to supporting (and not thwarting) learners’ basic psychological needs for autonomy, competence, and relatedness-described by Self-Determination Theory (SDT).
• Greater satisfaction (versus frustration) of these basic needs promotes more internalized forms of behaviour regulation, which for learners, tends to translate into better engagement, deeper learning, stronger academic performance, and greater psychological well-being.
• Being autonomy-supportive is not a trait but rather a skill. It can be learned and applied in many different settings in medical education, including in the classroom and in clinical learning environments.
• Engaging in autonomy-supportive teaching strategies is relatively simple, flexible, and mutually beneficial for learners and teachers alike.

## Notes On Contributors

Adam Neufeld, MD, MSc is a Family Medicine resident at the University of Calgary. His research interests are in medical education and positive psychology. ORCID ID: https://orcid.org/0000-0003-2848-8100
