# Supplementary material for: Autonomy-Supportive Teaching in Medicine: From Motivational Theory to Educational Practice
Source: MedEdPublish (2016). 2021 May 10;10:117. [Version 1] doi: 10.15694/mep.2021.000117.1 (PMC10939533; doi:10.15694/mep.2021.000117.1)
Supplement: Peer review history

# Reviewer report: Reviewer response for version 1

**DOI:** 10.21956/mep.18895.r26990

**Published:** 2021-10-14

**Authors:** Keith Wilson

**Affiliations:** 1 Dalhousie University

This review has been migrated. The reviewer awarded 5 stars out of 5 The author of this article makes a strong case for educators to take an autonomy-supportive approach to teaching in order to enhance learning and to help elevate intrinsic motivation. The article provides an overview of Self-Determination Theory (SDT) and a number of practical examples that can be applied in a number of teaching scenarios. The article is very well written and guides the reader through theory to practice using the frame of SDT. Of particular note is that this article focuses additionally on more challenging learning environments including teaching in clinical settings. The real strength of this paper lies in helping the reader not only what is good to do but also what to avoid and why. This has practical applications for creating a more supportive learning environment that will aid, not hinder, learner “engagement, performance and well-being.”This article should be widely read by teachers both in the classroom and in the clinical setting as well as institutional leaders. It has an excellent balance of theory and practical tips for any educator.

Reviewer Expertise:

NA

No decision status is available

# Reviewer report: Reviewer response for version 1

**DOI:** 10.21956/mep.18895.r26989

**Published:** 2021-10-13

**Authors:** Julie Hunt

**Affiliations:** 1 Lincoln Memorial University

This review has been migrated. The reviewer awarded 5 stars out of 5 This article, submitted as a practical tips article, reads much like a review of the literature on SDT and how it can be applied to medical education both in the classroom and in the clinic. The fundamental tenets described here are universally important to medical educators, and I recommend it as a read for all faculty in the healthcare professions who are not already familiar with SDT. The only improvement I can think of would have been the addition of a table of autonomy-supportive and controlling teaching behaviors for quick reference and to highlight those to the reader.

Reviewer Expertise:

NA

No decision status is available

# Reviewer report: Reviewer response for version 1

**DOI:** 10.21956/mep.18895.r26987

**Published:** 2021-06-03

**Authors:** Nneka Orakwue-Ononye

This review has been migrated. The reviewer awarded 5 stars out of 5 Great write up...very useful guide and reminder for medical educators. I like the SDT’s motivation continuum as makes for a quick visual summary. Also the need for feedback to be both ways between educator and learner is of utmost importance. Thank you!

Reviewer Expertise:

NA

No decision status is available

# Reviewer report: Reviewer response for version 1

**DOI:** 10.21956/mep.18895.r26988

**Published:** 2021-05-10

**Authors:** Haseeb Rizvi

This review has been migrated. The reviewer awarded 5 stars out of 5 A wonderful and well thought out overview of the self-determination theory and its application towards creating a better medical education experience. Autonomy based learning seems to be the future of medical education and more medical colleges need to integrate these ideas into the curriculums.

Reviewer Expertise:

NA

No decision status is available
